# An Experimental Study on the Effect of Temperature on Piezoelectric Sensors for Impedance-Based Structural Health Monitoring

**DOI:** 10.3390/s140101208

**Published:** 2014-01-10

**Authors:** Fabricio G. Baptista, Danilo E. Budoya, Vinicius A. D. de Almeida, Jose Alfredo C. Ulson

**Affiliations:** Faculdade de Engenharia, UNESP-Univ Estadual Paulista, Campus de Bauru, Departamento de Engenharia Elétrica, Av. Eng. Luiz Edmundo Carrijo Coube, 14-01, Bauru-SP 17033-360, Brazil; E-Mails: dbudoya.eng@gmail.com (D.E.B.); vnsdare@hotmail.com (V.A.D.A.); ulson@feb.unesp.br (J.A.C.U.)

**Keywords:** piezoelectric sensors, PZT, structural health monitoring, SHM, electromechanical impedance, EMI, temperature

## Abstract

The electromechanical impedance (EMI) technique is considered to be one of the most promising methods for developing structural health monitoring (SHM) systems. This technique is simple to implement and uses small and inexpensive piezoelectric sensors. However, practical problems have hindered its application to real-world structures, and temperature effects have been cited in the literature as critical problems. In this paper, we present an experimental study of the effect of temperature on the electrical impedance of the piezoelectric sensors used in the EMI technique. We used 5H PZT (lead zirconate titanate) ceramic sensors, which are commonly used in the EMI technique. The experimental results showed that the temperature effects were strongly frequency-dependent, which may motivate future research in the SHM field.

## 1. Introduction

Piezoelectric sensors have been intensively studied in recent years for an important and promising application, the structural health monitoring (SHM) of various types of structures, such as bridges and aircrafts. The main purpose of SHM systems is to monitor a structure and detect incipient damage, thereby increasing safety and reducing maintenance costs. Therefore, SHM systems are an important field of research from both scientific and industrial perspectives [1].

There are several nondestructive testing (NDT) methods [2] that are used to detect structural damage, such as Lamb waves [3,4], acoustic emission testing [5,6], comparative vacuum monitoring [7], and eddy current testing [8]. The electromechanical impedance (EMI) technique [9] is considered one of the most promising of these various methods, because it is very simple to implement and uses low-cost, small and lightweight piezoelectric sensors. These sensors consist of adhesive tapes bonded to the host structure that are minimally invasive and can be used in real time and in *in-situ* SHM systems.

This technique is simple to perform. When the sensor is bonded to the structure to be monitored, the piezoelectric effect produces an interaction between the mechanical impedance of the host structure and the electrical impedance of the sensor. Therefore, the mechanical condition of the host structure can be monitored simply by measuring and analyzing the electrical impedance of the piezoelectric sensor.

The EMI method has been studied for decades, and several studies [10,11] have shown that its use is feasible for lab-scale and complex structures; however, practical problems have hindered the efficient and reliable application of EMI to real-world structures. Temperature effects have been cited in the literature as one of the most critical and challenging of several practical problems. Here, we experimentally study the effect of temperature variations on the sensor impedance signatures. We present new and important results that may guide future research in this area.

This article is organized as follows: in Section 2, we introduce the basic principle of the EMI technique and discuss how structural damage is detected. A preliminary analysis of the temperature effects on the electrical impedance of the piezoelectric sensor is presented in Section 3. The experimental procedure for making measurements at different temperatures is presented in Section 4, and the experimental results are presented and discussed in Section 5. Finally, the paper ends with conclusions in Section 6 and a list of references.

## 2. EMI Method

In this section, we discuss the principle on which the EMI method is based and how structural damage is detected.

### 2.1. Underlying Principle

**Figure 1.**
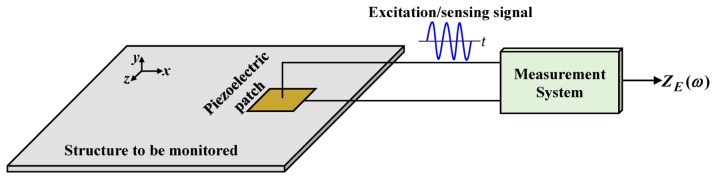
Underlying principle of the EMI method.

The EMI technique is based on the electromechanical interaction between the mechanical impedance of the structure to be monitored and the electrical impedance of the piezoelectric sensor that is bonded to the structure. The basic configuration is shown in Figure 1.

Figure 1 shows a square piezoelectric patch that is attached to the structure to be monitored. The measurement system simultaneously excites the patch through an excitation signal with an appropriate frequency (ω) and acquires the corresponding response signal that provides the electrical impedance (*Z_E_*(*ω*) of the patch. Thus, the piezoelectric device operates simultaneously as an actuator and a sensor in the EMI method. The electrical impedance signature is obtained in the same frequency range within which the excitation signal is generated.

The patch can be considered as a parallel plate capacitor, whereas the dielectric is a piezoelectric material. The excitation voltage produces an electric field, as well as a mechanical deformation, because of the piezoelectric effect. If the excitation signal voltage is sufficiently small, the piezoelectric effect is approximately linear. Using this consideration, the basic piezoelectric equations can be obtained from the Gibbs free energy [12] as follows:

(1) $$D_{m}=d_{m\beta }^{H,\theta }T_{\beta }+\varepsilon_{mk}^{T,H,\theta }E_{k}+m_{mk}^{T,\theta }H_{k}+p_{m}^{T,H}d\theta$$

(2) $$S_{\alpha }=s_{\alpha \beta }^{E,H,\theta }T_{\beta }+d_{\beta m}^{H,\theta }E_{m}+d_{\beta m}^{E,\theta }H_{m}+\alpha_{\alpha }^{E,H}d\theta$$

where *d_mβ_* and *d_βm_* are the piezoelectric constants, *m_mk_* are the magneto-dielectric constants, *p_m_* are the pyroelectric constants, *α_α_* are the thermal expansion coefficients, *H_k_* and *H_m_* are the magnetic field components, *θ* is the temperature, *S_α_* is the mechanical strain tensor, *T_β_* is the mechanical stress tensor, *S_αβ_* is the compliance tensor, *E_k_* and *E_m_* are the electric field components, *D_m_* are the electric displacement components, and *ε_mk_* is the dielectric permittivity tensor of the material. The superscripts *E*, *H*, *θ* and *T* show that the electric field, the magnetic field, the temperature, and the mechanical stress are constant. Considering the symmetry of the tensors, we have *α*, *β* = 1, 2, …, 6 and *m*, *k* = 1, 2, and 3.

If we neglect the effect of the temperature and the magnetic field, Equations (1) and (2) can be rewritten as:

(3) $$D_{m}=d_{m\beta }T_{\beta }+\varepsilon_{mk}^{T}E_{k}$$

(4) $$S_{\alpha }=s_{\alpha \beta }^{E}T_{\beta }+d_{\beta m}E_{m}$$

Equation (3) describes the direct piezoelectric effect by which the mechanical stress (*T_β_*) in the material causes an electric displacement (*D_m_*). In the reverse piezoelectric effect described by Equation (4), the application of the electric field (*E_m_*) causes a corresponding mechanical strain (*S_α_*).

Typically, the piezoelectric sensors used in the EMI method are very thin, with a thickness on the order of fraction of a millimeter. In this case, the deformation of the piezoelectric material in the direction of the thickness (*i.e.*, the *y*-axis in Figure 1) is very small (on the order of tens of nanometers) compared to the other directions and can be neglected. Moreover, for a one-dimensional (1D) problem, only the deformation of the material along the length of the monitored structure (*i.e.*, the *x*-axis in Figure 1) is considered. From these considerations, the electrical impedance of the piezoelectric patch for PZT (lead zirconate titanate) material is given by [13]:

(5) $$Z_{E}(\omega )=\frac{1}{j\omega C_{0}}\|jZ_{T}{\left(\frac{s_{11}}{d_{31}\ell }\right)}^{2}\left[\frac{1}{2}\text{tan}\left(\frac{k\ell }{2}\right)-\frac{1}{\text{sin}\left(k\ell \right)}+\frac{Z_{S}}{j2Z_{T}}\right]$$

where *Z_E_*(*ω*) is the electrical impedance, *ω* is the angular frequency, *C*_0_ is the static capacitance for a square patch of size *ℓ*, *k* is the wave-number, *Z_T_* is the mechanical impedance of the piezoelectric patch, *Z_S_* is the mechanical impedance of the monitored structure, *d*_31_ is the piezoelectric constant, *s*_11_ is the compliance at a constant electric field, ∥ indicates a parallel connection, and *j* is the unit imaginary number.

Equation (5) shows that the electrical impedance (*Z_E_*(*ω*)) of the piezoelectric sensor depends on the mechanical impedance (*Z_S_*) of the monitored structure. Any change in the mechanical impedance of the host structure caused by damage, such as cracks and corrosion, will result in a corresponding variation in the electrical impedance of the piezoelectric sensor. Therefore, the structural health can be evaluated simply by measuring and analyzing the electrical impedance signatures of the sensor.

In the past, impedance measurements have been performed using commercial impedance analyzers, such as the 4192A and 4194A from Agilent Technologies (Santa Clara, CA, USA) and Hewlett-Packard (Palo Alto, CA, USA). These instruments provide highly accurate measurements but are expensive, slow and have many features that are not required in the EMI method. To overcome these drawbacks, several alternative measurement systems have been developed [14–16] to enable fast and low-cost data acquisition. Typically, the damage is identified and quantified by comparing two electrical impedance signatures using metric indices. We present this procedure in the next section.

### 2.2. Damage Characterization

Basic damage characterization is accomplished using appropriate metric indices. The most widely used indices are the root mean square deviation (RMSD) and the correlation coefficient deviation metric (CCDM). These indices compare two electrical impedance signatures, where one of the signatures is acquired when the structure is considered to be healthy and is used as a reference, which is commonly called the baseline.

The RMSD index is based on a Euclidean norm [17] and is given by:

(6) $$\text{RMSD}=\sum_{k=\omega_{I}}^{\omega_{F}}\sqrt{\frac{{\left[Z_{E,D}(k)-Z_{E,H}(k)\right]}^{2}}{{Z^{2}}_{E,H}(k)}}$$

where *Z_E,H_*(*k*) and *Z_E,D_*(*k*) are the electrical impedance signatures (*i.e.*, the magnitude, the real part or the imaginary part) that are given by Equation (5) for the structure under healthy and damaged conditions, respectively, and are measured at a frequency *k* that ranges from *ω_I_* (the initial frequency) to *ω_F_* (the final frequency).

The CCDM index is based on the correlation coefficient [18] and is given by:

(7) $$\text{CCDM}\;=\;1-C_{C}$$

where *C_C_* is the correlation coefficient calculated using the following equation:

(8) $$C_{C}=\frac{\overset{\omega_{F}}{\underset{k=\omega_{I}}{\text{∑}}}\left[Z_{E,H}(k)-{\bar{Z}}_{E,H}\right]\;\left[Z_{E,D}(k)-{\bar{Z}}_{E,D}\right]}{\sqrt{\overset{\omega_{F}}{\underset{k=\omega_{I}}{\text{∑}}}{\left[Z_{E,H}(k)-{\bar{Z}}_{E,H}\right]}^{2}}\sqrt{\overset{\omega_{F}}{\underset{k=\omega_{I}}{\text{∑}}}{\left[Z_{E,D}(k)-{\bar{Z}}_{E,D}\right]}^{2}}}$$

where *Z_EH_*(*k*) and *Z_E,D_*(*k*) are the signatures as defined above, and *Z̄_E,H_* and *Z̄_E,D_* are the averaged signatures under healthy and damaged conditions, respectively, that are computed over a frequency range between *ω_I_* (the initial frequency) and *ω_F_* (the final frequency).

The RMSD and CCDM indices exhibit different behaviors. The RMSD index is more sensitive to variations in the amplitude of the electrical impedance signatures. On the other hand, the CCDM index is more sensitive to changes in the shape of the signatures, such as frequency shifts. However, both indices are significantly affected by temperature variations. The effects of temperature on the electrical impedance of the sensor and the indices are analyzed in the next section.

## 3. Temperature Effects

Equations (1) and (2) show that the piezoelectric sensors are also pyroelectric and magnetoelectric devices, that is, the electric displacement (*D*) from the direct piezoelectric effect and the mechanical strain (*S*) from the reverse piezoelectric effect are dependent on the magnetic field (*H*) and the temperature (*θ*). However, the effects of the magnetic field and the temperature are commonly neglected in deriving electromechanical models for the EMI method, as was assumed in deriving Equation (5).

The effect of the magnetic field can be safely neglected in most applications; however, the same is not true for temperature effects, because the piezoelectric sensors used in the EMI method are also significantly pyroelectric. Thus, temperature changes cause corresponding variations in the electrical impedance of the sensor. Therefore, the temperature can be determined by measuring the electrical impedance of the piezoelectric sensors [19].

Damage detection is accomplished by comparing two electrical impedance signatures that are obtained at different times: therefore, temperature changes cause variations in the RMSD index. Consequently, the RMSD index can be high even for a healthy structure, leading to a false positive diagnosis.

On the other hand, the CCDM index is insensitive to variations in the amplitude of the electrical impedance and is only sensitive to variations in the shape of the impedance signature. Therefore, if we only consider variations in the amplitude of the electrical impedance resulting from temperature changes, the CCDM index would be a good option for avoiding false diagnoses of the monitored structure from temperature effects. However, the natural frequencies (*i.e.*, the resonance peaks in the impedance signatures) of the structure are temperature-dependent. The natural frequencies decrease as the temperature increases [20]. These variations in the natural frequencies cause frequency shifts in the electrical impedance signatures.

**Figure 2.**
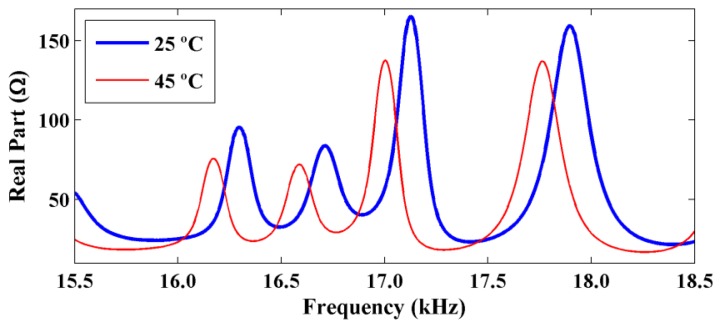
Variations in the amplitude and frequency shifts in real part of the electrical impedance resulting from temperature changes.

Figure 2 shows two electrical impedance signatures that were obtained at temperatures of 25 °C and 45 °C for an aluminum beam with dimensions of 500 mm × 38 mm × 3 mm using a 5H PZT patch with dimensions of 15 mm × 15 mm × 0.267 mm.

Figure 2 shows variations in both the amplitude and frequency of the electrical impedance signatures. Consequently, the CCDM index varies with the temperature even for healthy structures. Thus, temperature effects must be compensated for to ensure proper functioning of impedance-based SHM systems.

Several methods have been developed to compensate for temperature variations. Lim *et al.* [21] developed a new damage detection technique using data normalization that was based on kernel principal component analysis (KPCA) to improve detectability and minimize false positive diagnoses from temperature variations. However, as with artificial neural networks (ANN), the developed technique requires a large amount of training data for proper operation. Otherwise, false diagnoses can occur if the training data does not include a specific condition or temperature range.

Bastani *et al.* [22] developed a compensation technique using a piezoelectric sensor array. In this method, a sensor array and statistical parameters are used to identify changes caused by damage, temperature and environmental vibrations. The frequency of excitation is very high: therefore, structural damage has a significant effect on the closest sensor response unlike environmental change conditions, which affect all the sensors almost uniformly regardless of where they are located on the structure. Thus, the effects of damage can be identified from environmental effects on electrical impedance signatures. However, this method requires a large amount of sensors for effective operation, and the structural damage must be near one of the sensors. Hong *et al.* [23] developed a similar technique using a hybrid damage-monitoring scheme, where an accelerometer sensor was used to perform global damage monitoring (which were associated with temperature effects), and local damage monitoring was performed using the conventional EMI method.

Therefore, the techniques presented above are particularly appropriate and efficient for specific applications and conditions. Simple techniques for general applications can be obtained using modified versions of RMSD and CCDM metric indices. Sun *et al.* [24] used the cross-correlation between the baseline and the updated signatures to compensate for the frequency shifts. Park *et al.* [25] compensated for both the frequency and the magnitude shifts using a modified RMSD metric index. Koo *et al.* [26] modified the method developed by Park *et al.* [25] to develop an effective frequency shift (EFS) to compensate for temperature effects. The method was based on shifting the updated impedance signature relative to the baseline signature to maximize the correlation coefficient. This method has continued to be used in recent studies. Yun *et al.* [27] used the EMI technique to detect damage in a bridge and combined the EFS method with ANN to compensate for temperature effects.

Although the EFS method is simple, its efficiency decreases if a wide frequency band is used to compute the damage metric indices. The experimental results in this paper show that the frequency shift is not constant, but increases with the excitation frequency, requiring narrow frequency band.

## 4. Experimental Setup

To investigate how temperature affects the sensor impedance signatures, we performed several experiments with an aluminum beam with dimensions of 500 mm × 38 mm × 3 mm and a mass of 179.552 g. A 5H PZT patch with dimensions of 15 mm × 15 mm × 0.267 mm was bonded approximately 30 mm from the end of the specimen using cyanoacrylate glue appropriate for high temperatures.

**Figure 3.**
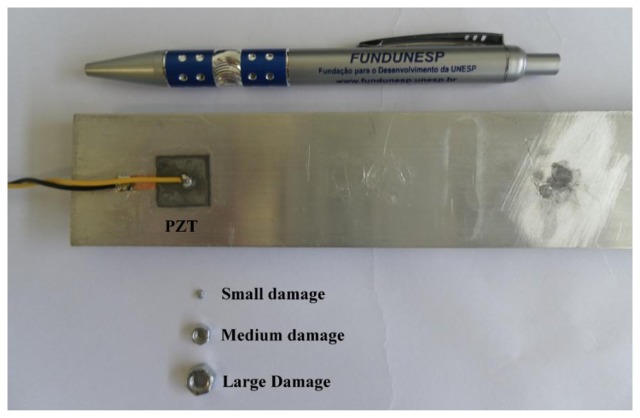
Aluminum beam with the PZT patch and damage of different intensities.

Structural damage of different intensities was induced in the structure by placing a small nail-head (representing a low damage level) with dimensions of 2 mm × 1 mm and a mass of 0.029 g, a medium steel nut (representing a medium damage level) with dimensions of 5 mm × 2 mm and a mass of 0.312 g, and a large nut (representing a large damage level) with dimensions of 8 mm × 4 mm and a mass of 0.988 g, all at a distance center-to-center of 100 mm from the sensor. The mass loadings of the monitored structure were approximately 0.016%, 0.17%, and 0.5%, respectively, for the low, medium and large damage levels. The mass loading produced variations in the mechanical impedance of the structure and could consequently be related to the structural damage. Figure 3 shows the aluminum beam with the PZT patch, the nail-head, and the nuts.

The measurement of the electrical impedance of the sensor was performed using a system [28] based on a multifunction data acquisition (DAQ) device, LabVIEW, and a personal computer (PC). The DAQ used in the tests was a NI USB-6361 with a sampling rate of 2 MS/s (samples/second), and the sensor was excited using a chirp signal with amplitude of 1 V in a frequency range of 0–550 KHz with a frequency step of 2 Hz. The temperature of the specimen was measured with a K-type digital thermocouple. We varied the temperature of the whole specimen including the PZT patch from 25 to 102 °C using a hot gun. Although the temperature stability provided by a heat gun is not as good as that of a chamber, the measuring system [28] provides fast measurements (about 5 s for a frequency range of 0–550 kHz), allowing its use satisfactorily.

**Figure 4.**
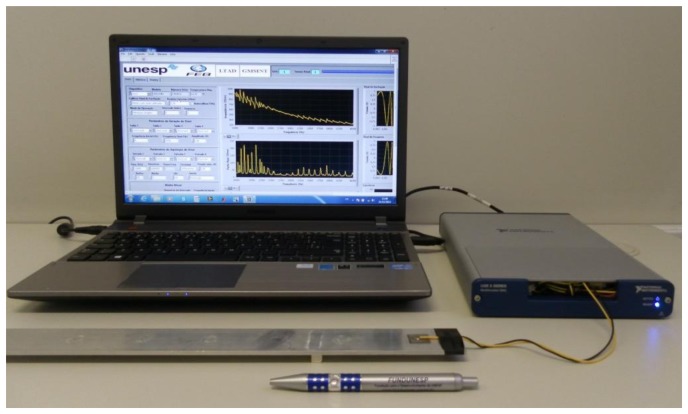
Experimental configuration.

In addition to measuring the electrical impedance, we measured the capacitance of the PZT patch over the same temperature range. Equation (5) shows that the piezoelectric sensor is predominantly capacitive with a static capacitance *C*_0_. Thus, it was important to check how the capacitance varied with the temperature. The capacitance was measured with a simple multimeter. Figure 4 shows the experimental configuration.

All the measurements were obtained by supporting the specimen on its ends using rubber blocks on a desk.

## 5. Experimental Results and Discussion

We have organized the results into three sections. In the first section, we estimate the sensitivity of the system to detect structural damage by calculating the RMSD and CCDM indices for damage with different intensities induced in the aluminum beam. Next, the effects of the temperature on the electrical impedance signatures of the sensor are analyzed in detail. Finally, in the last section, the frequency shifts in the electrical impedance signatures are compensated for by maximizing the correlation coefficient, where we verify that the efficiency of this method decreases as the frequency band increases.

### 5.1. Damage Detection Sensitivity

The structural damage was quantified by calculating the RMSD and CCDM indices in an appropriate frequency band. As is well known, frequencies below 100 kHz, particularly in the 0–50 kHz band, produce higher damage indices than other frequency bands. These results have been theoretically and experimentally analyzed in previous studies [29,30] and experimentally confirmed in the present study. However, it is critical to choose an appropriate frequency band and some types of structures or damage may require higher frequencies.

**Figure 5.**
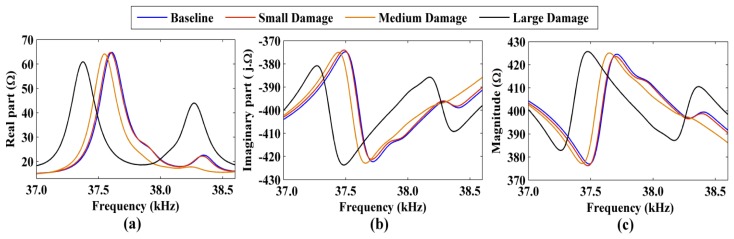
Variation in (**a**) the real part; (**b**) the imaginary part; and (**c**) the magnitude of the impedance signatures for damage of different intensities.

Therefore, the RMSD and CCDM indices were calculated over a 16–40 kHz frequency band and reasonable results were obtained, such that *ω_I_* = 16 kHz and *ω_F_* = 40 kHz in Equations (6) and (8). Figure 5 shows the electrical impedance signatures (*i.e.*, the real part, the imaginary part, and the magnitude) that were obtained for the healthy structure (baseline signatures) and the damaged structure by placing the nail-head and the nuts at a distance of 100 mm from the piezoelectric sensor. Only a narrow frequency band of 37–38.6 kHz is shown to facilitate the comparison of the impedance signatures.

Figure 5 shows that the damage caused variations in the electrical impedance signatures relative to the baseline signatures, *i.e.*, structural damage was present. As expected, the large damage level caused significant variations in the impedance signatures compared to the low damage level.

**Figure 6.**
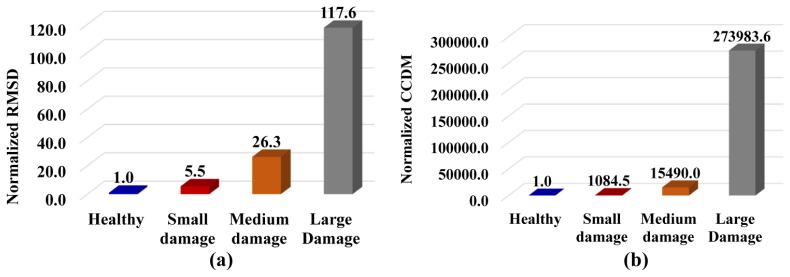
Normalized (**a**) RMSD and (**b**) CCDM indices for different damage intensities.

The identification and quantification of damage was simply evaluated using the RMSD and CCDM indices calculated in Equations (6) and (7), respectively, as shown in Figure 6. The indices were calculated using the real part of the impedance signatures, which has been reported as being more sensitive to damage detection [13]. Both indices were normalized, resulting in RMSD = 1 and CCDM = 1 for the healthy structure.

The results showed that the system exhibited a high sensitivity for detecting structural damage. Although the nail-head induced a low damage level (*i.e.*, only approximately 0.016% of the mass loading), the CCDM index was 1,084.5 times higher than that obtained for the healthy structure. The values obtained for the medium and large damage levels were 15,490.0 and 273,983.6 times higher, respectively, than the value obtained for the healthy structure. Lower values of the RMSD index of 5.5, 26.3 and 117.6 were found for the low, medium and large damage levels, respectively.

These results are important for estimating the sensitivity of the system to detect damage as well for identifying parameters to evaluate the potential of false positive diagnoses of structural health from temperature effects. Temperature effects are analyzed in the next section.

### 5.2. Temperature Effects

**Figure 7.**
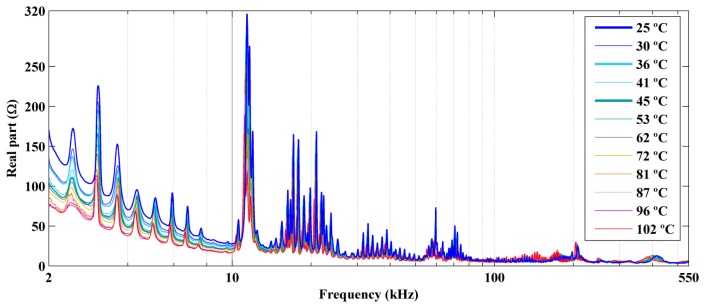
Real part of the impedance signatures obtained at different temperatures.

We performed experiments for temperatures ranging from 25 °C to 102 °C. Figures 7 and 9 show the real part, the imaginary part, and the magnitude of the electrical impedance signatures, respectively, over a frequency range of 2–550 kHz. The frequency axis is shown on a logarithmic scale to facilitate comparison of the data. Frequencies below 2 kHz are not shown, because the impedance in this frequency band was very high and would make data comparison more difficult.

The figures show decreases in the amplitude and frequency shifts of the real part, the imaginary part and the magnitude of the impedance signatures. These variations can be analyzed in detail by choosing narrower frequency bands. Figures 10 and 12 show the real part, the imaginary part and the magnitude of the impedance signatures corresponding to resonance peaks at frequencies of 5.91 kHz, 20.95 kHz, and 197.80 kHz, respectively, at a temperature of 25 °C.

The three resonance peaks clearly exhibited left frequency shifts as the temperature increased. However, these shifts were not constant at a given temperature but increased with the frequency of the resonance peak. Therefore, the frequency shifts depended on both the temperature and the frequency. The figures show that increasing the temperature from 25 °C to 102 °C produced a frequency shift of Δ*f* = −134 Hz relative to the impedance signature at 25 °C for the resonance peak at 5.91 kHz. The frequency shifts for the resonance peaks at 20.95 kHz and 197.80 kHz were Δ*f* = −450 Hz and Δ*f* = −4,300 Hz, respectively, for the same temperature variation.

In addition, the real part, the imaginary part, and the magnitude of the impedance signatures exhibited the same resonance peaks and frequency shifts, although the peaks were more prominent for the real part and resulted in higher damage indices, as shown in Figures 7 and 6, respectively.

All the frequency shifts (Δ*f*) at different temperatures that are shown in Figures 10 and 12 are reported in Table 1. Once again, the temperature of 25 °C and the resonance peaks obtained at this temperature were used as references. A clear decreasing trend of the resonance peaks (*i.e.*, a left frequency shift, which corresponded to a negative Δ*f*) was observed as the temperature increased. The frequency shifts were more significant for the resonance peaks at higher frequencies.

Figure 13 shows the frequency shifts (Δ*f*) relative to the impedance signature obtained at 25 °C as a function of the frequency for temperatures ranging from 30 °C to 102 °C.

In addition to being dependent on the temperature, the left frequency shift (*i.e.*, the negative Δ*f*) also consistently increased as the frequency of the impedance signatures increased from 2 kHz to 200 kHz. The variation in Δ*f* was approximately linear at low frequencies up to 15 kHz, where the slope depended on the temperature variation: however, this analysis was not conclusive. In this case, the frequency shift (Δ*f*) was approximately 0.3% of the frequency of the resonance peak for a temperature variation of +5 °C (relative to that at a temperature of 25 °C). For a temperature variation of +77 °C, Δ*f* was significantly higher at approximately 2% of the resonance peaks at low frequencies.

For frequencies above 200 kHz, deformations in the impedance signatures were observed, especially at high temperatures. There were no significant resonance peaks at frequencies below 2 kHz that could enable the frequency shifts to be accurately estimated.

In addition to the frequency shifts in the impedance signatures, variations in the amplitude of the impedance signatures were also observed. Equation (5) shows that the piezoelectric sensors are predominantly capacitive devices with a capacitance *C*_0_. Therefore, it was important to analyze the variation of the capacitance with temperature. Figure 14 shows how the capacitance varied for temperatures between 26 °C and 67 °C.

**Figure 8.**
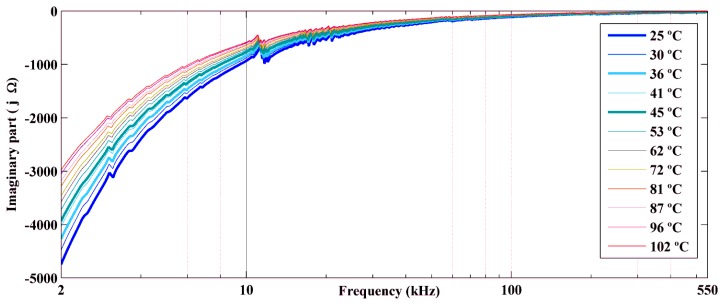
Imaginary part of the impedance signatures obtained at different temperatures.

**Figure 9.**
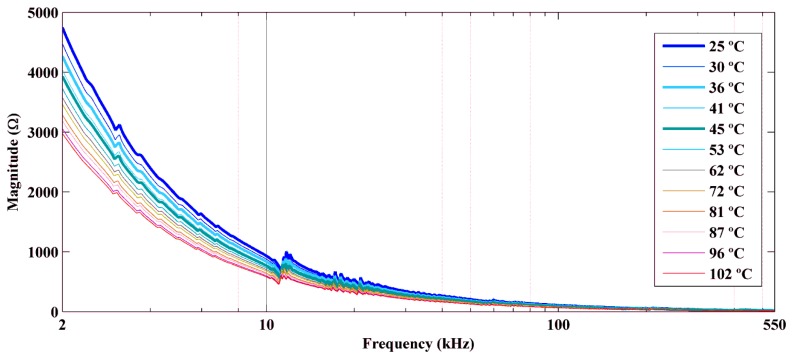
Magnitude of the impedance signatures obtained at different temperatures.

**Figure 10.**
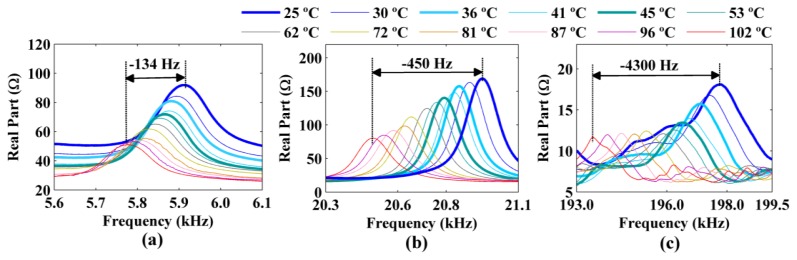
Frequency shifts of the real part of the impedance signatures for resonance peaks at (**a**) 5.91 kHz; (**b**) 20.95 kHz; and (**c**) 197.80 kHz considering the temperature at 25 °C as a reference.

**Figure 12.**
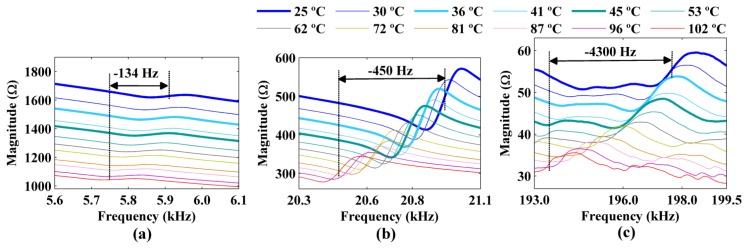
Frequency shifts of the magnitude of the impedance signatures for resonance peaks at (**a**) 5.91 kHz, (**b**) 20.95 kHz, and (**c**) 197.80 kHz considering the temperature at 25 °C as a reference.

**Figure 13.**
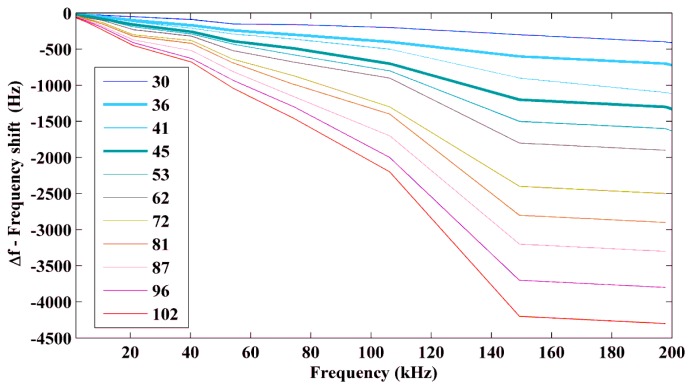
Frequency shift (Δ*f*) as a function of the temperature and the frequency.

**Figure 14.**
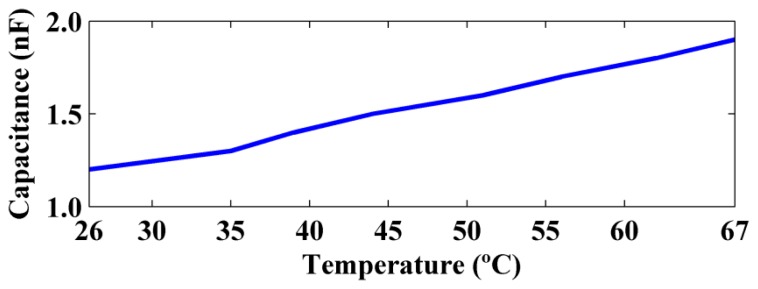
Capacitance as a function of the temperature.

Figure 14 shows that the capacitance varied approximately linearly with the temperature. The capacitance increased from 12 nF to 19 nF when the temperature increased from 26 °C to 67 °C. Therefore, there was a significant variation of 58.3% of the capacitance. This result was consistent with the decrease in the amplitude of the impedance signatures, because a higher capacitance results in a lower reactance, as was observed for the imaginary part of the impedance shown in Figure 8. The effect of the temperature on the correct diagnosis of structural health is analyzed in the next section.

### 5.3. Compensation for Temperature Effects and False Diagnoses

As previously mentioned, the EFS is a very simple method of compensating for temperature effects, which can be applied to a wide variety of structures under diverse conditions. This method consists of shifting the current impedance signature (after temperature variation) relative to the baseline (reference) impedance signature to maximize the correlation coefficient.

Therefore, before computing the CCDM index, the current impedance signature *Z_E,D_*(*k*) must be continuously shifted using a loop routine until the maximum correlation coefficient *C_C_* (ideally, *C_C_* = 1) is obtained. In Section 5.2, we showed that increasing the temperature produced a left frequency shift (*i.e.*, a negative Δ*f*) of the resonance peaks of the impedance signature. Thus, the current impedance signature had to be shifted in the opposite direction (*i.e.*, right-shifted) to maximize the correlation coefficient, as shown below:

(9) $$\begin{array}{ll} Z_{E,D_{\left(i\right)}}(k)=Z_{E,D_{\left(i-1\right)}}(k-d_{f}), & \Delta f<0 \end{array}$$

where *d_f_* is the frequency step provided by the measurement system, and the subscript (*i*) denotes the number of the iteration. In this study, measurements were taken at *d_f_* = 2 Hz. The frequency shift in Equation (9) had to be performed using a sufficient number of iterations (*i*) to obtain the maximum correlation coefficient in Equation (8). Compensating for temperature effects in a healthy structure is expected to result in *C_C_* ≌ 1.

On the other hand, the impedance signature for a decrease in the temperature must be shifted to the left, as shown below:

(10) $$\begin{array}{ll} Z_{E,D_{\left(i\right)}}(k)=Z_{E,D_{\left(i-1\right)}}(k+d_{f}), & \Delta f>0 \end{array}$$

Despite the simplicity and feasibility of this approach for a wide variety of structures, the results are only satisfactory if a narrow frequency band (*ω_I_*−*ω_F_*) is used to calculate the correlation coefficient in Equation (8). This limitation arises because the frequency shift (Δ*f*) of the resonance peaks is not constant over the entire frequency range but increases with the frequency. Thus, shifting the current impedance signature (*Z_E,D_*(*k*)) using Equation (9) or (10) only results in a good match with the baseline impedance signature (*Z_E,H_*(*k*)) over a narrow frequency band.

**Figure 15.**
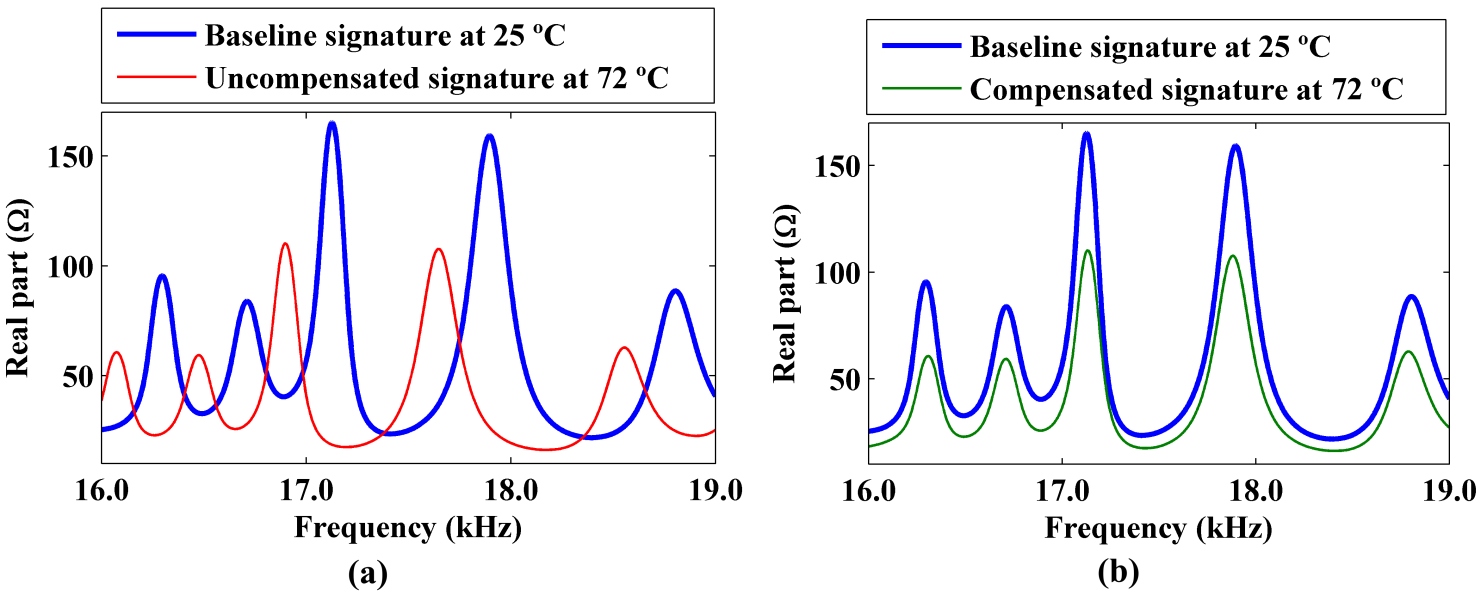
(**a**) Uncompensated and (**b**) compensated impedance signature obtained at 72 °C relative to the baseline signature at 25 °C using a narrow frequency band.

Figure 15 shows the uncompensated and compensated real part of the impedance signature that was obtained at 72 °C relative to the baseline signature obtained at 25 °C using a narrow frequency band of 16–19 KHz.

Figure 15b shows that there was a good match between the compensated impedance signature and the baseline signature over a narrow frequency band of 16–19 kHz, because the five resonance peaks in this band exhibited approximately the same frequency shift (Δ*f*). However, the match between the two impedance signatures could be unsatisfactory over a wider frequency band.

**Figure 16.**
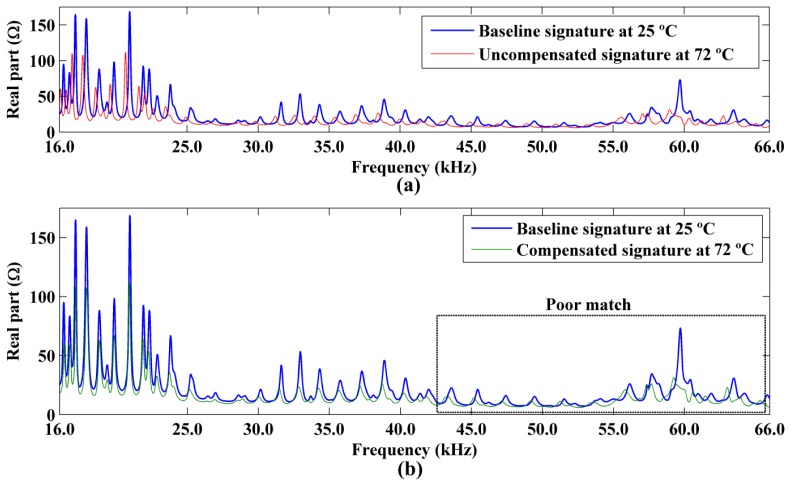
(**a**) Uncompensated and (**b**) compensated impedance signature obtained at 72 °C compared to the baseline signature at 25 °C using a wide frequency band.

For example, Figure 16 shows a similar analysis over a wide frequency band of 16–66 kHz. Figure 16b shows a good match between the two signatures for frequencies between 16 kHz and approximately 23 kHz. However, the match between the two signatures was unsatisfactory for frequencies ranging from approximately 43 kHz to 66 kHz, because the frequency shifts (Δ*f*) of the resonance peaks at high frequencies were significantly higher than those at low frequencies.

Consequently, the poor match between the two impedance signatures resulted in a low correlation coefficient. Thus, the maximum correlation coefficient (*C_C_*) that was obtained using this method of compensation decreased as the frequency band (*ω_I_*−*ω_F_*) increased.

**Figure 17.**
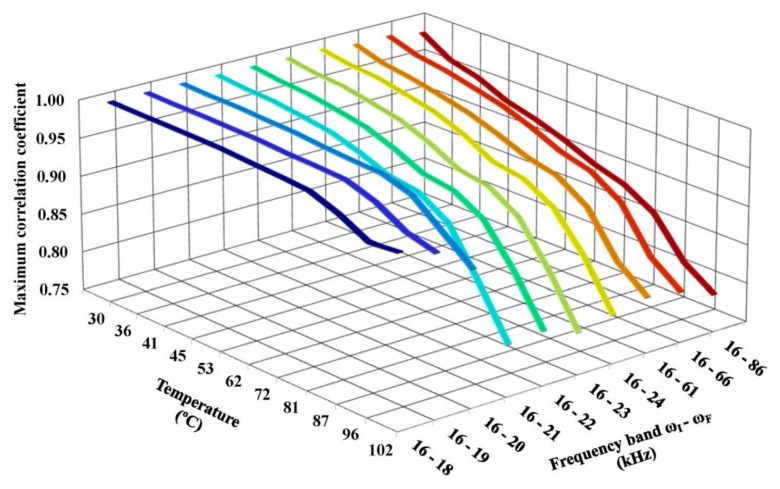
Maximum correlation coefficients obtained using the compensation method.

Figure 17 shows the maximum correlation coefficient that was obtained using this method of compensation as a function of the temperature and the frequency band. The results were obtained by adopting the impedance signature obtained at 25 °C as the baseline. The coefficients were calculated using the real part of the impedance, which produced more accurate results.

The maximum correlation coefficient clearly decreased as both the temperature and frequency band increased. For example, for a narrow frequency band of 2 kHz (*ω_I_* = 16 kHz and *ω_F_* = 18 kHz), the correlation coefficient was approximately 1.0 and ranged from 0.999 (at a temperature of 30 °C) to 0.958 (at a temperature of 102 °C). However, using a wide frequency band resulted in a significantly lower correlation coefficient. For example, for a wide frequency band of 70 kHz (*ω_I_* = 16 kHz and *ω_F_* = 86 kHz), the correlation coefficient was lower and ranged from 0.985 (at a temperature of 30 °C) to 0.783 (at a temperature of 102 °C).

Equation (7) shows that a low correlation coefficient produces a high CCDM index, which could result in false positive diagnoses of the monitored structure. Note that incipient damage causes very small variations in the correlation coefficient on the order of thousandths or less. Therefore, the frequency band chosen in the procedure to compensate for temperature effects can significantly impact the correct diagnosis of the structure to detect low damage levels. In this case, it is difficult to distinguish temperature effects from the effects of damage.

**Figure 18.**
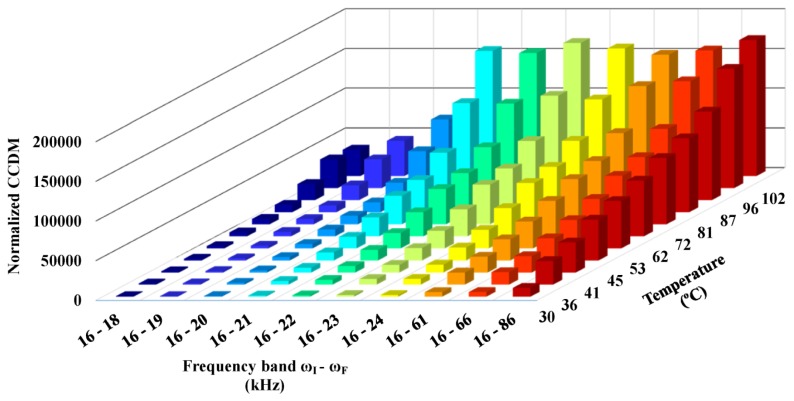
Normalized CCDM indices obtained for the healthy structure after compensating for temperature effects.

Figure 18 shows the normalized CCDM indices that were obtained for the healthy structure after applying the compensation method for temperatures ranging from 30 °C to 102 °C and using different frequency bands (*ω_I_*−*ω_F_*) in Equation (8). Again, the real part of the impedance signature that was obtained at 25 °C was used as the baseline.

Figure 18 shows that the CCDM index increased with the frequency band (with some exceptions). In some cases, the CCDM indices were higher than those obtained for low and medium damage levels induced in the structure (see Section 5.1) even after temperature effects were compensated for. Only the three CCDM indices obtained at 30 °C over the frequency bands of 16–18 kHz, 16–19 kHz, and 16–20 kHz were lower than the CCDM index obtained for the low damage level induced by the nail-head (which was 0.016% of mass loading).

These results show that a frequency band must be sufficiently narrow for the frequency shift method to effectively compensate for temperature effects and to avoid false positive diagnoses, particularly for detecting low damage levels. On the other hand, wider frequency bands may include more resonance peaks and, consequently, increase the sensitivity of the system to detect structural damage, especially incipient damage. Therefore, the width of the frequency band is a critical issue for compensation techniques based on the frequency shift.

## 6. Conclusions

In this study, we investigated the effect of temperature on the electrical impedance signatures of a conventional 5H PZT sensor used in structural health monitoring. The variations in both the amplitude and the frequency were analyzed experimentally by using an aluminum specimen and obtaining impedance signatures at temperatures ranging from 25 °C to 102 °C.

The experimental results showed that the variations in the amplitude of the impedance signatures were related to the temperature-dependence of the capacitance of the piezoelectric sensor. In addition, the frequency shifts of the resonance peaks that resulted from temperature variations were not constant over the entire frequency range but increased with the frequency. Thus, the frequency band used to calculate the damage indices played an important role in compensating for temperature effects by maximizing the correlation coefficient. The results showed that a sufficiently narrow frequency band must be used to avoid a false positive diagnosis of the monitored structure.

Therefore, temperature effects are a critical problem for structural health monitoring based on electromechanical impedance, especially in detecting low damage levels, and efficient compensatory methods for temperature effects remain to be developed.

## Figures and Tables

**Figure 11.**
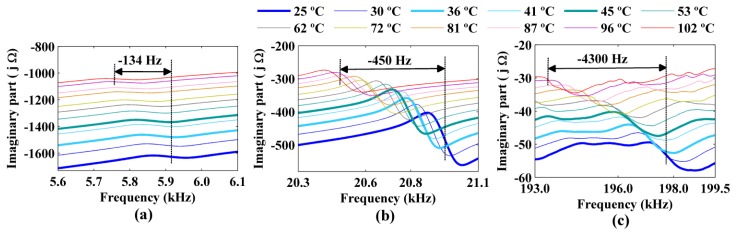
Frequency shifts of the imaginary part of the impedance signatures for resonance peaks at (**a**) 5.91 kHz; (**b**) 20.95 kHz; and (**c**) 197.80 kHz considering the temperature at 25 °C as a reference.

**Table 1.** Frequency shifts (Δ*f*) for three resonance peaks at different temperatures.

	**Peak at 25 °C (kHz)**	**Temperature (°C)**										

		**30**	**36**	**41**	**45**	**53**	**62**	**72**	**81**	**87**	**96**	**102**
**Δ*f* (Hz)**	**5.91**	−18	−30	−36	−46	−56	−70	−84	−92	−106	−120	−134
	**20.95**	−50	−100	−120	−160	−190	−230	−300	−320	−370	−410	−450
	**197.80**	−400	−700	−1,100	−1,300	−1,500	−1,900	−2,500	−2,900	−3,300	−3,800	−4,300
